# Utilization of modern contraceptives and predictors among women in Shimelba refugee camp, Northern Ethiopia

**DOI:** 10.1371/journal.pone.0212262

**Published:** 2019-03-06

**Authors:** Aselefech Seyife, Girmatsion Fisseha, Henock Yebyo, Gebreamlak Gidey, Hadgu Gerensea

**Affiliations:** 1 Araya Kahsu College of Health Science, Nursing School, Axum, Ethiopia; 2 Department of Public Health, College of Health Science, Mekelle University, Mekelle, Ethiopia; 3 Department of Midwifery, College of Health Science and Referral Hospital, Aksum University, Aksum, Ethiopia; 4 School of Nursing, College of Health Science and Referral Hospital, Aksum University, Aksum, Ethiopia; USC Keck School of Medicine, Institute for Global Health, UNITED STATES

## Abstract

Women living in refugee camps, in addition to the common hardships, such as drought, and famine, are also prone to another peculiar problem: an unintended pregnancy. The impact of unintended pregnancy is so severe that the rate of women who die or suffer an injury while giving birth in crisis settings is almost double the world average death rate. Thus, this study was aimed to investigate the utilization of modern contraceptive and associated factors among women in the reproductive age group in Shimelba refugee camp, Northern Ethiopia. A community-based cross-sectional study was employed and 329 study subjects were selected using simple random sampling technique with a face-to-face interview. The prevalence of using modern contraceptive was 47.7% and the study showed that being older [AOR = 0.017, 95%CI: 0.001, 0.467], being single [AOR = 0.17, 95%CI:0.031,0.914], being unemployed [AOR = 0.21, 95% CI:0.001,0.392], having no partner support [AOR = 0.006, 95% CI:0.001,0.044], and inconvenient service site AOR = 0.089,95% CI:0.013, 0.595] were factors that contributed to women not using modern contraceptive methods. Receipt of counseling on family planning utilization was more likely to helps women to use it [AOR = 3.37, 95% CI: 1.1095, 10.236]. Our study concluded that the current prevalence rate of contraceptive use is fairly good. However, much effort has to be made to improve this result. The situations in refugee can exacerbate the existing barriers to the use of contraceptives. Given its grave consequence on the livelihood of women, the contraceptive issue should be given due emphasis using several techniques including education to expand the awareness on modern contraceptive so as to augment access to family planning.

## Introduction

Family planning is the ability of couples or individuals to decide both their desired number of children and the space of time between their children through the use of contraceptive methods. There are several cost-effective contraceptive methods which provide important health and human right benefits [1]. The use of contraceptive methods is allowed to anyone who needs it including refugee women and men to avoid unwanted pregnancy [2].

By nature, most refugee camps are a risk for sexual violence. Particularly women and children are exposed to rape and its consequences: unintended pregnancy, unsafe abortions, and spread of STI/HIV. To make things worse, displaced peoples mostly have broken social structure and women are the most affected part of the societal influence of this problem [3–10]. These problems, in the long term, can inflict a psychological problem and mortality on the affected women [3].

Currently, the numbers of refugees in the world are increasing. The UNHCR estimates around 15.4 million border-crossing refugees are found in the world. Among those, females accounted for 48% of the refugee population. By hosting-refugees, Kenya leads in Africa by accommodating 564,900 refugees, and Ethiopia, with 376,400 refugees, was ranked the six largest refugee hosting African country by the end of 2012 [4–5].

A study in African conflict-affected area affecting women showed that 30%-40% of women needed to space births & 12%-35% to limit their births [6]. But, there are different factors that influence their expectations, perceived needs and demand like the situation in the refugee’s country of origin and host country condition [7].

There are several factors which preclude women not to get the contraceptives they needed. The consequences of lack of contraception put one in five women at risk for unwanted pregnancy and complications including unsafe abortion which accounts for 78% of all maternal mortality among refugee women [8–9].

Report from UNHCR indicated that in Djibouti, Kenya, & Uganda refugee camps, contraceptive utilization ranges from 5.1%-14.6% [7, 11–13], a much lower than other settlements such as in Jordan and Malaysia which range 21.4%-34.2% [14–15]. However, adequate data on the utilization of modern contraceptive and associated factors among women in Ethiopian refugee camps is not still identified. Therefore, the current study is mainly focused on the magnitude and barriers of contraceptive utilization in Ethiopia, shire site, refugee camps.

## Methods

### Study settings

The study was conducted in Shimelba Refugee Camp, Tigray regional state, Northern Ethiopia from February up to March 2014. Shimelba refugee camp is one of the big camps used for settling Eritrean refugees since 2004. According to the 2014 report of Administration for Refugees Returnees Affairs (ARRA), around 5,935 Eritrean refugees live in the camp. Almost half of these are females with 1164 of them being in a reproductive age group. This population is being served by only one health center, funded by the UNHCR.

### Study design and sampling

Community-based cross-sectional study design was used. The study participants were sexually active women in the reproductive age group (15–49 years age). The sample size was calculated using a single population proportion formula by taking a 14.6% of modern contraceptive utilization from Uganda refugee camp [16] with 95% confidence interval and 4% marginal error.

Adding a 10% expected number of non-respondent or refusals, the total sample size was 329 women. Out of the list obtained from ARRA, women were randomly selected for the study using the lottery method.

### Measurement

Data were collected using interview by taking standard questionnaire which was adapted from CDC for reproductive health assessment toolkit for conflict-affected women [17]. For Data quality, the English questionnaire was translated into local language, Tigrigna, and again re-translated back into English by another translator to check the consistency of original meaning. To decrease interviewer biases, we thoroughly trained the data collectors on the objective, methods, and ethics prior to data collection.

The data were collected by four data collectors and one supervisor. Modern contraceptive methods utilization was the outcome variable. The modern contraceptive methods were oral contraceptives pills, IUD, male and female condoms, implants, injectables, emergency hormonal contraception, tubal ligation and vasectomy. If a woman or male partner would be using any of the following contraceptive methods during the study period, she was considered a modern contraceptive user.

Knowledge of women on modern contraceptive was measured based on the answered number of questions in the standard questionnaire. If she answered at least 80% of the standard knowledge questions, she would be grouped as “high” knowledge. If she answered 60–70%, she would be grouped in “moderate” knowledge, and if she answered below 60%, she would be grouped in “low” knowledge group making the women to be poorly informed about modern contraceptive methods. Similarly, the attitude of the mothers was defined by the mean score of the attitude questions. As such, mothers with a score of attitude questions less than the mean were considered as with “good” attitude.

### Data analysis

Data were entered into SPSS 16 for windows. The data were screened for errors and cleaned away. Then, they were categorized into convenient groups where it is appropriate, and checked for some assumptions prior to data analysis. The univariate analyses were computed using descriptive statistics. To identify factors that may be associated with the utilization of modern contraceptive methods, we employed multivariate logistic regression. The effect sizes of the predictors in the sample were depicted using Odds Ratio (OR) and that of the population were estimated using 95% confidence interval of OR. An effect with P-value less than 5% was considered statistically significant.

## Ethical consideration

Ethical approval was obtained from the research ethical committee (REC) and institutional review board (IRB) of Mekelle University, College of Health Sciences. Permission was also received from ARRA main office, Addis Ababa. Oral consent was taken from the respondents for their willing participation in the study if their age was greater than18 and oral consent was taken if age was less than 18 years from guardians or close family members. No personal identifiers were decoded and reported in the final report.

## Results

### Socio-demographic characteristics

The participation was 100% all women were interviewed. A slightly higher number of women (57.8%) came from rural areas of Eritrea. The women stayed in Shimelba camp from 7 to 14 months. The average age of the women was 27 years. Two-third of the women had attended a formal education. On average, each woman had 2.9 children (+1.95) (Table 1).

**Table 1:** Socio-demographic characteristics of women in reproductive age group in Shimelba, Northern Ethiopia, 2014 (n = 329).

Variables	Number	Percent
**Age of women (n = 329)**		
15–19	56	17
20–24	86	26.1
25–29	72	21.9
30–34	41	12.5
35–39	39	11.9
40–49	35	10.6
**Ethnicity (n = 329)**		
Tgriagna	115	35
Kunama	205	62.3
Saho	9	2.7
**Marital status (n = 329)**		
Married or living with partner	303	92
Single	14	4.3
Divorced	9	2.7
Windowed	3	1
**Educational status of respondents (n = 329)**		
Can’t read and right	98	29.8
Can read and right	16	4.9
1-8th grade	167	50.7
Secondary and above	48	14.6
**Occupation of respondents (n = 329)**		
House wives	251	76.3
Employed	16	4.9
Merchant	11	3.3
Students	38	11.5
Daily labor	13	4
**Previous place of residence (n = 329)**		
Urban	139	42.2
Rural	190	57.8
**Duration in the camp (month) (n = 329)**		
Jul-60	181	55
61–120	56	17
121–168	92	28
**Partner age (n = 303)**		
20–29	101	33.3
30–39	114	37.6
40–80	88	29.1
**Occupation of partners (n = 303)**		
No Job	176	58.1
Employed	38	12.5
Merchant	54	17.8
Students	22	7.3
Daily labor	13	4.3

### Knowledge and attitude of women about modern contraceptive methods

The majority of the respondents (95.4%) had heard at least one type of modern contraceptive methods during their lifetime. While majority of the participants (95.1%) had heard about short term contraceptive methods, a little less than half the participants (45.3%) had heard about long-term contraceptive methods and yet very few (10%) had heard about permanent contraceptive methods. In addition, 275(83.6%) of participants had an awareness of at least one source where modern contraceptive methods are available. Among the women who had awareness on contraceptive methods, about 145(46.2%) women had low knowledge, 76(24.2%) had moderate knowledge and 93(29.6%) women had high knowledge.

Regarding the level of attitude, 171 (52%) had a negative attitude towards the use of modern contraceptive methods. While 80% of the total women agreed that pregnancy is a risk for women’s life, 40% favored having many children. With regard to beliefs and myths, 23% of participants believed that taking contraception may induce infertility, and 22% of the respondents believed in that taking contraception can cause a birth defect.

### Magnitude of modern contraceptive use

**Fig 1:**
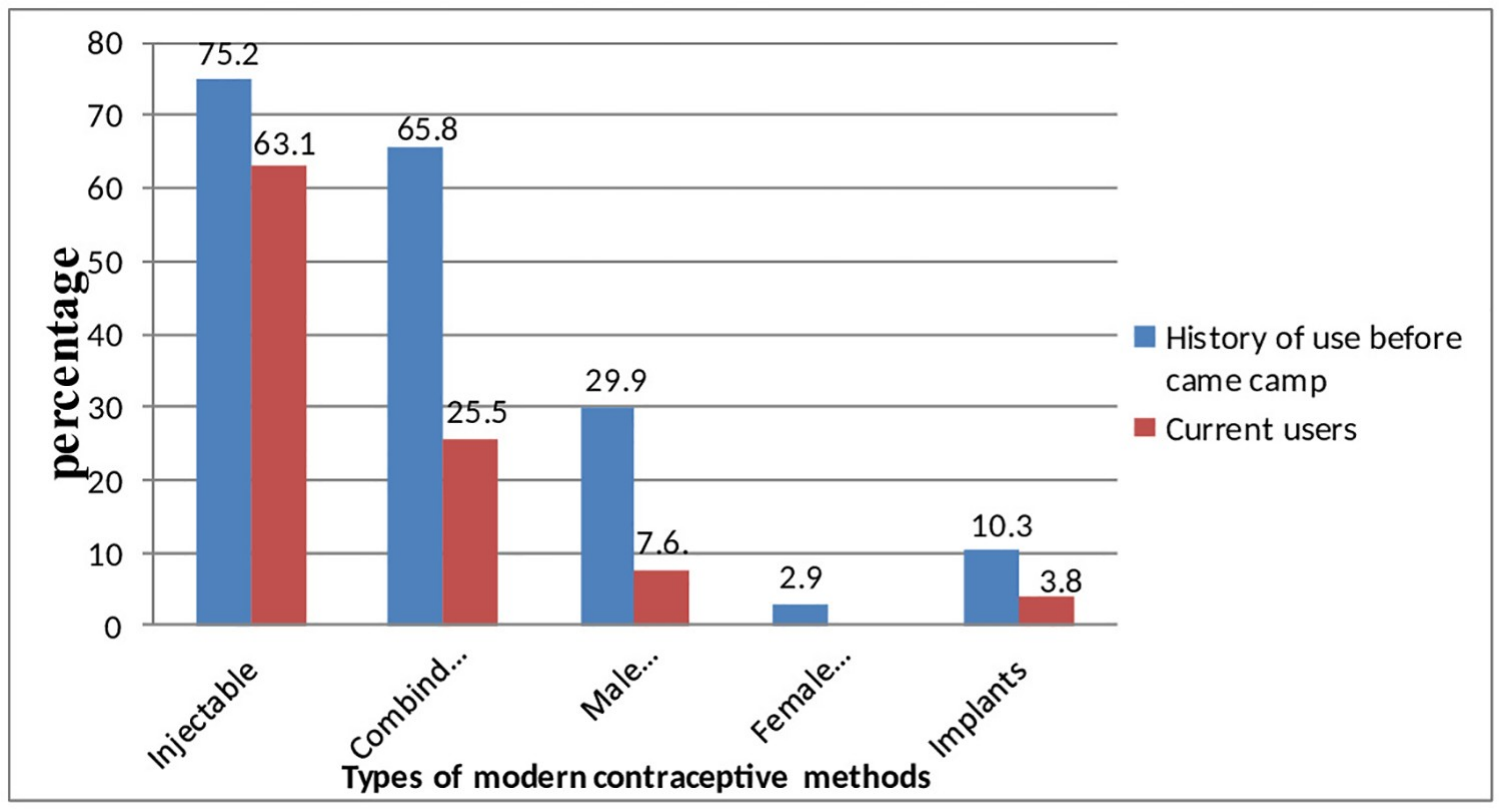
Prevalence of modern contraceptive methods use among women of reproductive age group (N = 329), Shimelba refugee camp, Northern Ethiopia, 2014.

157(47.7%) have used modern contraceptive with the most utilized contraceptive being injectables 99(63.1%) followed by combined oral contraceptive pills 40(25.5%), male condom 12(7.6%) and implant 6(3.8%). None of the respondents had ever used a female condom, IUCD and permanent methods (Fig 1).

Most of them (77.7%) were using contraceptives for birth spacing and only few were using contraceptive for birth limiting (16.6%). The main reasons for deterring mothers not to use contraceptive methods are the beliefs and myths (45%) inconvenience of service hours (33.7%), the inconvenience of service sites (39.8%) and fear of religious rules (39%). All the mothers received the contraceptive methods only from the health center serving them.

### Role of partners in using modern contraception

Among the total 303 respondents who had a regular partner during the study period, 48.5% of respondents reported that they hadn’t discussed contraceptive use with their partners. As such, almost half of the women decided to use modern contraceptive use only on their own decision. (Table 2).

**Table 2:** Role of partner in modern contraceptive methods utilization, Northern Ethiopia, 2014 (n = 303).

Variables	Frequency	Percent
**Partner’s approval the use of modern contraception (303)**		
Yes	162	53.5
No	141	46.5
**Discussion with partner on the use of FP (303)**		
Yes	156	51.50%
No	147	48.5
**Partner involvement in contraceptive use (303)**		
Yes	122	40.3
No	181	59.7
Way of partner involvement (122)*		
By reminding appointment day	95	77.9
By bringing contraception	4	3.3
By reminding time of use	22	18
By using by himself	17	13.9
**Responsible for decision making on the use of FP (303)**		
Partner only	31	10.2
Women only	145	47.9
Women & partner jointly	127	41.9

### Factors associated with modern contraceptive utilization

After controlling for the effect of other variables, the odds of using modern contraception in women of age range 40–49 was 98.3%, which was less than those in the age range of 15–19[AOR = 0.017,95% CI: 0.001,0.467]. On the other hand, women who are single were less to use modern contraception as compared to married or cohabited women (AOR = 0.17,95% CI:0.031,0.914). In terms of job possession, housewives were 79% less likely to use modern contraceptive methods as compared to their counterparts who had an occupation of daily labor [AOR = 0.21, 95% CI: 0.001, 0.392].

The odds of contraception use was 91% less among those women who used to make the decision to use contraceptive methods jointly with their partners as compared to mothers with the partner-dominated decision [AOR = 0.087, 95%CI: 0.011, 0.708]. The use of modern contraception was 91% less among women for whom the service site was not convenient than the mothers in which the current site was convenient to them [AOR = 0.089, 95%CI: 0.013, 0.595]. The odds of using modern contraception was 3.37 times more for women who had gotten counseling services as compared to those who did not get [AOR = 3.37, 95% CI:1.1095, 10.236] (Table 3).

**Table 3:** Factors associated with modern contraceptive methods utilization among women in reproductive age in Shimelba refugee camp, Northern Ethiopia, 2014, n = 329.

Variables	MODERN CONTRACEPTION USE		OR(95%)CI	
	YES	NO	Crude	Adjusted
	No (%)	No (%)		
**Age in Years**				
15–19	15(26.8)	41(73.2)	1	1
20–24	42(48.8)	44(51.2)	2.61 (1.261, 5.398)	0.544(0.102, 2.898)
25–29	41(56.9)	31(43.1)	3.62(1.702, 7.678)	0.313(0.05, 1.966)
30–34	26(63.4)	15(36.6)	4.74(1.989, 11.287)	0.3(0.034, 2.651)
35–39	22(56.4)	17(43.6)	3.537(1.488, 8.411)	0.265(0.034, 2.091)
40–49	11(31.4)	24(68.6)	1.253(0.496, 3.165)	0.017(0.001,0.467)*
**Marital status**				
Ever married	141(55.1)	115(44.9)	1	1
Single	16(21.9)	57(78.1)	0.229(0.125, 0.42)	0.17(0.31, 0.914)*
**Woman occupation**				
House wife	122(48.6)	129(51.4)	0.591(0.188,1.856)	0.21(0.001,0.392)*
Employed	9(56.2)	7(43.8)	0.804(0.181, 3.557)	0.035(0.001,0.914)*
Merchant	8(72.7)	3(27.3)	1.667(2.94,9.445)	0.117(0.002,5.732)
Student	10(26.3)	28(73.7)	0.223(0.059,0.844)	0.144(0.004,5.136)
Daily labor	8(61.5)	5(38.5)	1	1
**Main decider on # children**				
Partner only	26(51)	25(49)	0.705(0.372, 1.334)	0.191(0.36, 1.024)
Woman only	40(39.6)	61(60)	0.444(0.266, 0.743)	0.169(0.42,0.677)*
Jointly	90(59.6)	61(40.4)	1	1
**Knowledge on modern contraceptive methods**				
High knowledge	61(65.6)	32(34.4)	3.3(1.918, 5.7)	0.932(0.255, 3.406)
Moderate knowledge	43(56.6)	33(43.4)	2.3 (1.29, 3.98)	1.799(0.539, 6.0)
Low knowledge	53(36.6)	92(63.4)	1	1
**Attitude regarding use modern contraception**				
Positive attitude	90(57)	68(43)	2.054(1.323, 3.189)	1.202(0.429,3.367)
Negative attitude	67(39.2)	104(60.8)	1	1
**Conveniences of FP unit**				
Yes	142(71.7)	56(28.3)	1	1
No	15(11.5)	116(88.5)	0.051(0.27,0.95)	0.089(0.013,0.595) *
**Getting counseling**				
Yes	115(66.1)	59(33.9)	5.244(3.267,8.418)	3.37(1.109,10.236)*
No	42(27.1)	113(72.9)	1	1
**Partner involvement**				
Yes	118(96.7)	4(3.3)	1	1
No	38(21)	143(79)	0.009(0.003,0.026)	0.006(0.001,0.044)*
**Main decider on modern contraception use**				
Only partner	14(45.2)	17(54.8)	1	1
Only woman	49(33.8)	96(66.2)	0.62(0.282,1.361)	0.442(0.073,2.673)
Jointly	93(73.2)	34(26.8)	3.321(1.479,7.46)	0.087(0.011,0.708)*

Key:

* indicates Statistically significant at P<0.05

## Discussion

The prevalence of modern contraceptive utilization in this study was 47.7% and the main factors affecting modern contraceptive utilization were age, relationship status, current work of women, main partner deciding on the number children in the family, conveniences of family planning service unit at the refugee camp, history of counseling for contraception, partner involvement, The main decider to use contraceptive in the family.

The magnitude of current modern contraceptive utilization in the study area of this research (47.7%) was higher when it compared with the UNHCR studies in five refugee camps, Djibouti (5.1%), Kenya (6.8%), Jordan (21.4%), Malaysia (34.2%) and in Uganda (14.6%) [7, 12–15]. This discrepancy could be due to the difference in sample size between the studies, accessibility of health services, lack of formal training for professionals, language barrier and cultural gaps. This result is also higher than the prevalence of modern contraceptive use in the Sub-Saharan African countries (21%), the Ethiopian national contraceptive use prevalence rate for total population (20%) and for currently married (27%). Moreover, the extent of modern contraceptive use was higher than their country of origin Eritrea, where contraception rate was 8% [18–20].

In our study, the preferred contraceptives were mainly short-term contraceptive methods which were injectables (63.1%), combined oral contraceptive (25.5%) male condom (7.6%) and implant (3.8%). This finding was similar with the finding in Djibouti (56.5%) and Uganda (63.2%) in which women uses injectables contraceptive in similar proportion. On the contrary, this study finding is different from Somalian refugees in Kenya and Burmese refugees in Malaysia where, oral contraceptive uptake rate was higher than other methods, 41.9% and 37% respectively[12, 15]. This difference may be due to the better availability of oral contraceptives in the refugee camp, Malaysia, where women had an access to obtain oral contraceptive easily from the pharmacy and hence can purchase from shops as compared for women in Shimelba refugee camp; the health center is the only option to obtain any type of modern contraception.

In this study, only 3.8% of women were currently using implants. Similar study result was found in Uganda [13]. In our study, none of the women used IUCD. However, in a study by UNHCR in Malaysia and Uganda refugees, women were using IUCD and permanent methods of contraception [13, 15]. This difference may due to the availability of varieties of contraception methods in the health facility of the refugee camps.

In this study, about 78% of women use modern contraceptives to space births rather than limit their births (16.6%). This is due to the reason that they have a high desire to have a child in the future (79.3%) which was corroborated by the finding that out of the respondents 71.3% had a desire to have a baby after two years. In addition to this, the majority of participants from none users had a negative attitude towards the use of modern contraceptive methods and a large proportion of women under the study had low knowledge of modern contraceptive methods. This finding is supported by another study outside the refugee camp in Mekelle city in which 65% of women use contraceptives for birth spacing and 17% for birth limiting [21].

In our study, about 39% of married women were under the age of 18 years of age. Despite the challenges in the camp life, 46.8% of women have three or more children; which indicates the value of having many children in the community. This is also reflected by the non-contraceptives users where a desire to have an additional child was their main reason for being none users (39%). In addition, religious prohibition (36%) and partner opposition (16%) had a significant contribution which is similar with the finding of a study in Somali and Uganda refugee women [7, 13].

The use of modern contraceptives is a significantly influenced by the age of women; the odds of using modern contraceptive methods among women greater than the age of 40 was 98.3% less likely when compared to women in 15–19 age group. This finding was different from a study among Pakistan refugee women where the odds of using contraception 1.06 times higher among women greater than the age of 35 as compared to other age groups. In addition to this, the study in Nepal shows that the odds of modern contraception use were 9 times more likely for women in the age of 35 as compared to those in 20–34 years of age [22–23]. The discordant result could be due to the difference in a study design and study subject. The study in Nepal was limited for women who were married but the scope of this study was for all women in a reproductive age which increase the probability of getting younger women in the study.

In this study, the occupational status of women significantly influenced the use of modern contraception. Housewives were 79% less likely to use modern contraceptive methods as compared to daily labor. This finding is supported by analysis in six different ethnic groups in Nepal which showed significantly increased contraception use among women who had the occupation as compared to those who had no job [23]. This might be due to the reason that this section of respondents spent most of their time on their professional carrier which, as a consequence, will decrease the desire for giving birth. In addition, women who were engaged in different jobs will have the probability of sharing information and experience about modern contraception with their colleagues than the housewives.

The issue of modern contraception is not a responsibility left to one partner alone; rather it needs the involvement of both partners. In this study, women whose partners were not involved in the use of contraception were 99% less likely to use modern contraception as compared to women whose partners were involved. This finding is supported by a study in Tigray and Hawasa [24–25].

The convenience of family planning services site, like distance, is a facility related factor which affects the use of modern contraception. In this study, the use of modern contraception was 91% less among women for whom the service site was not convenient as compared to women for whom the service site was convenient. This is supported by the finding that inconvenience of family planning services site, was the main reason among none-user adolescents, for their being none user. The same result was found among Ugandan refugees where family planning service was provided under the maternity ward which was not convenient for adolescents to use the services [13].

As a limitation of this study, the inclusion of all women in the reproductive age group without checking a sexual relationship may cause a problem since we don’t get the list of women in reproductive age who are with male partner or married. We did this by taking experience from UNHCR studies in refugee’s camps which took reproductive age women only. Other possible limitation is taking male partner condition only from the women may overestimate or underestimate some variables. So, only taking data from women side without interviewing partner could have affected the magnitudes. Moreover, the use of community health worker in the camp as data collectors could have overestimated the prevalence since they are involved in the health-related activities of the refugees. To minimize its effect, we tried to use data collector outside the camp but since the site was far away and away from the main road, It was difficult to get locally residing data collectors. Hence, it is better to consider these limitations in the interpretation of this finding.

## Conclusion

In this study, the prevalence of modern contraceptive use was high with the most preferred method being the short-acting family planning rather than long-acting. The main reasons that impedes the use of contraceptives are a desire to have more child, religious prohibition and inconvenience of services site. Generally the use of modern contraceptive methods are positively associated with obtaining of FP counseling service and negatively associated with being old in age, single in marital status, being unemployed (being housewives), lack of partner involvement, women-only decision maker and inconvenient service site. In addition, availability of multiple choices of contraceptive methods can foster family planning service. Different promotion activities using IEC materials should be prepared and distributed to the community on contraceptive messages; if possible, prepared by their own local language. Empowering women to engage into different forms of job is another important strategy. Services-site rearrangement and maintaining confidentiality by the provider is should be taken into account. Even though contraceptives, sometimes have side effects. They are very helpful in saving lives, spacing births, postponing child-bearing and preventing unintended pregnancies, etc.

## Supporting information

S1 Dataset (XLS)
